# Supplementary figures and images for: Patterns of molecular evolution in a parthenogenic terrestrial isopod (Trichoniscus pusillus)
Source: PeerJ. 2024 Jul 23;12:e17780. doi: 10.7717/peerj.17780 (PMC11276757; doi:10.7717/peerj.17780)

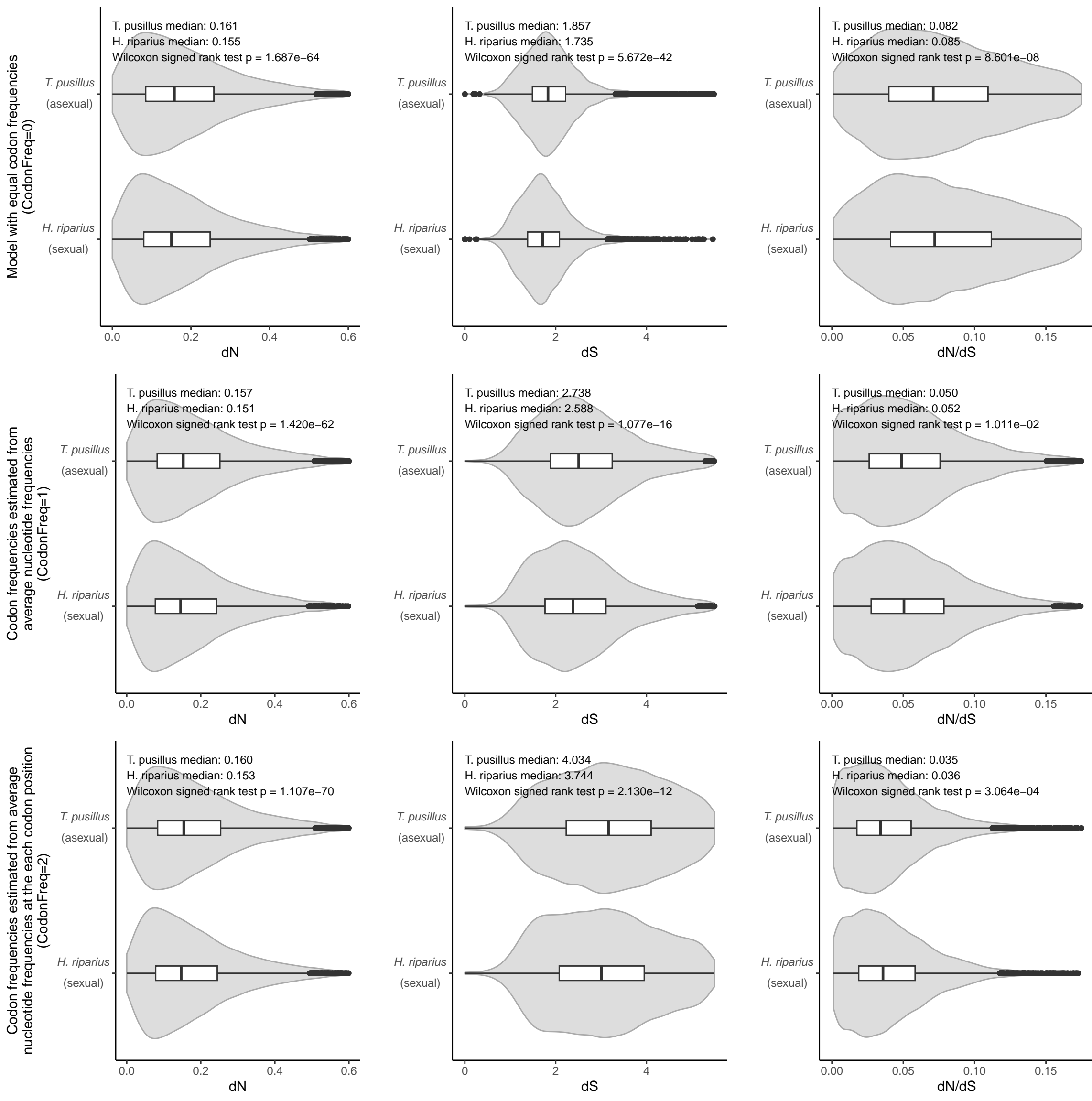

Supplement: Supplemental Information 1 — Violin plots of dN, dS, and dN/dS ratios, resulting from pairwise comparisons between T. pusillus vs. T. rathkei, and H. riparius vs. T. rathkei, using different codon models in the codeml package in PAML. [file peerj-12-17780-s001.pdf]

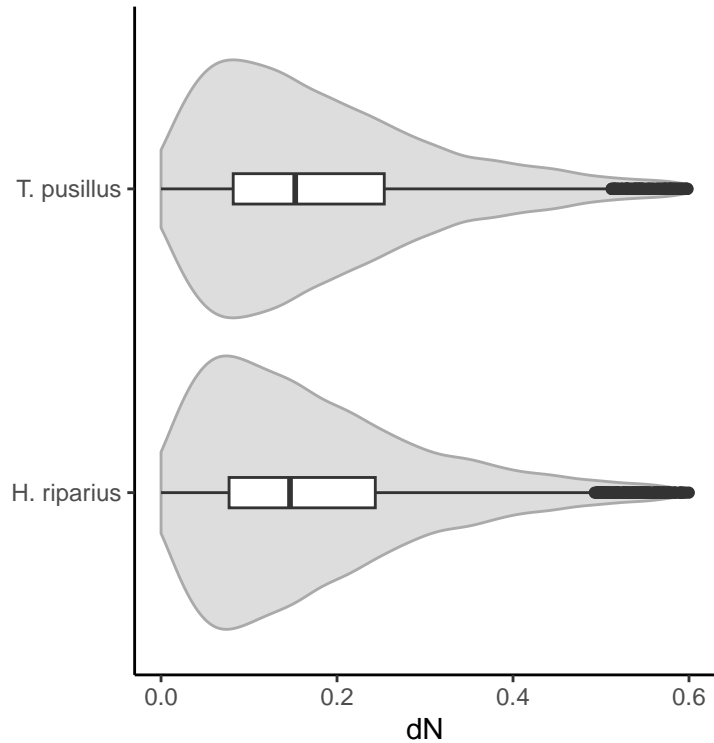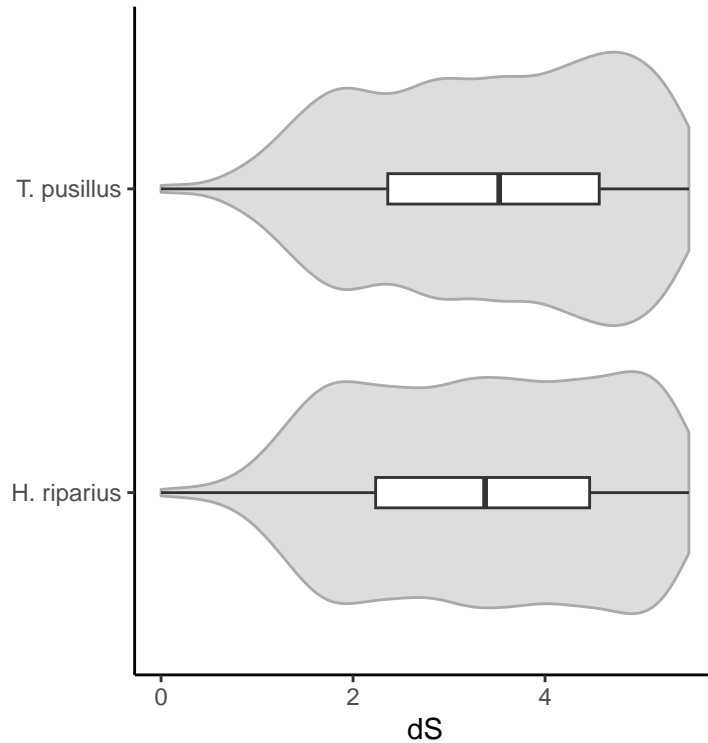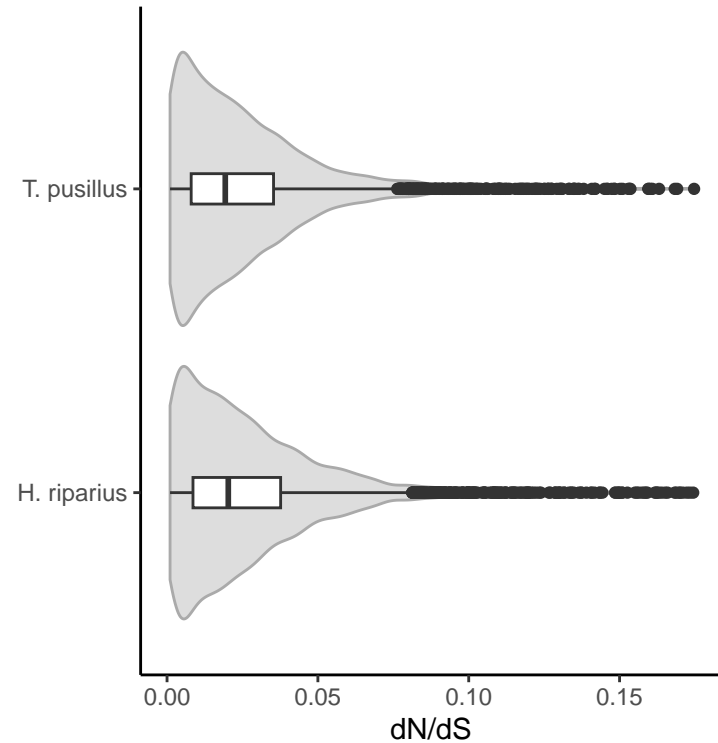

Supplement: Supplemental Information 2 [file peerj-12-17780-s002.zip › Yarbrough_Chandler_Code/6_orthofinder_dnds/Figure2.pdf]

*T. pusillus*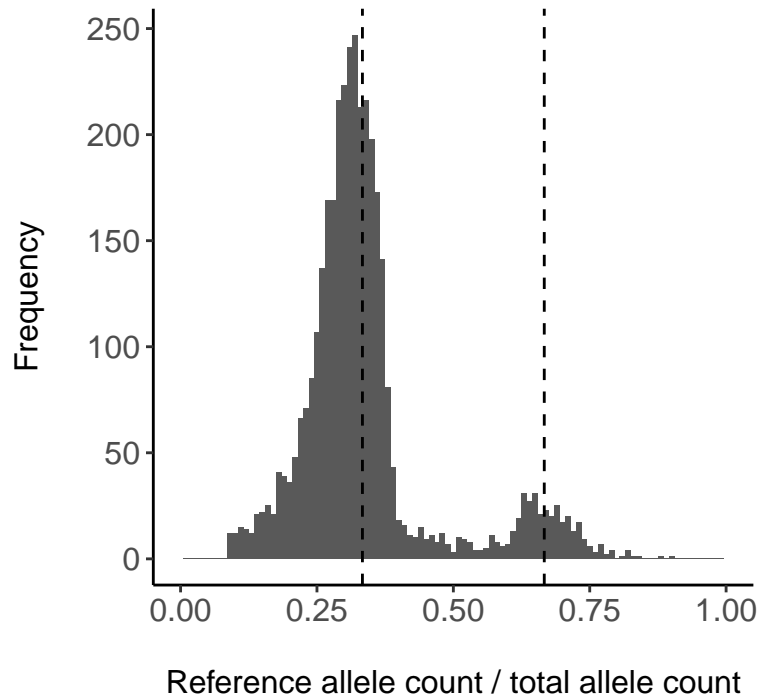*H. riparius*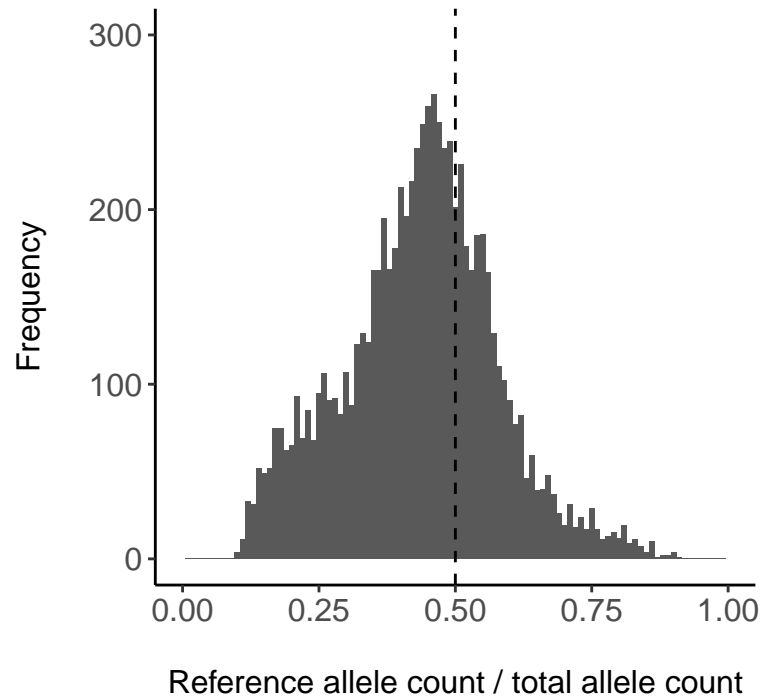*T. rathkei*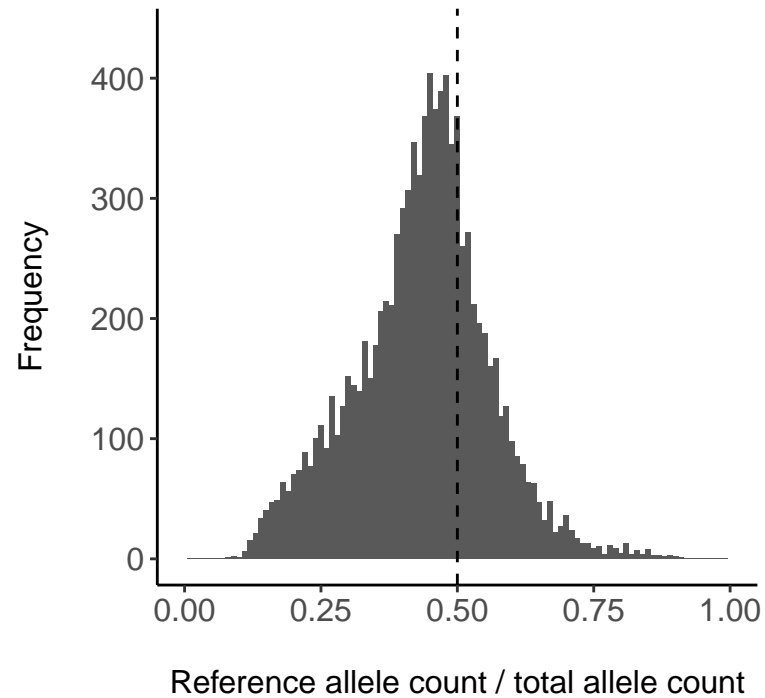

Supplement: Supplemental Information 2 [file peerj-12-17780-s002.zip › Yarbrough_Chandler_Code/5_snp_effects/5B_snp_similarity_and_allele_ratios/Figure1.pdf]

*T. pusillus*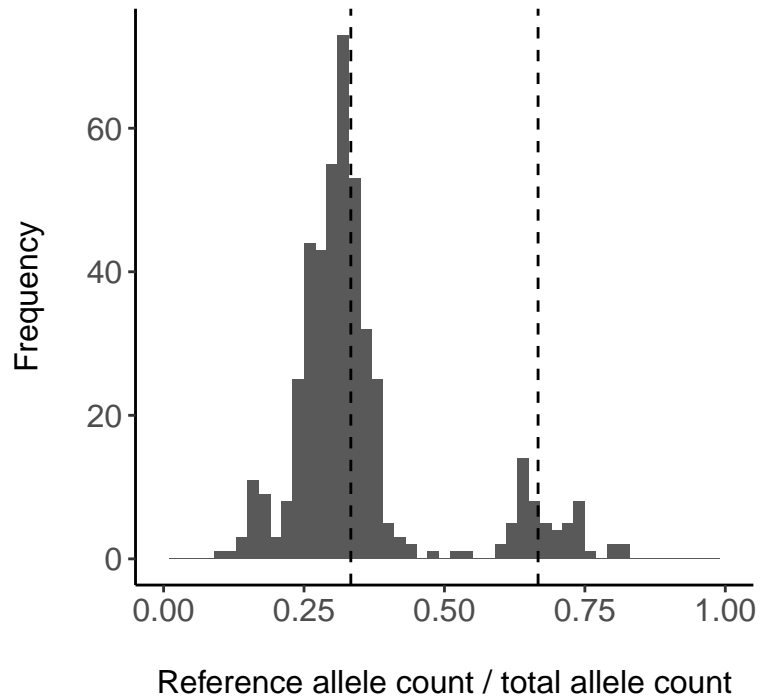*H. riparius*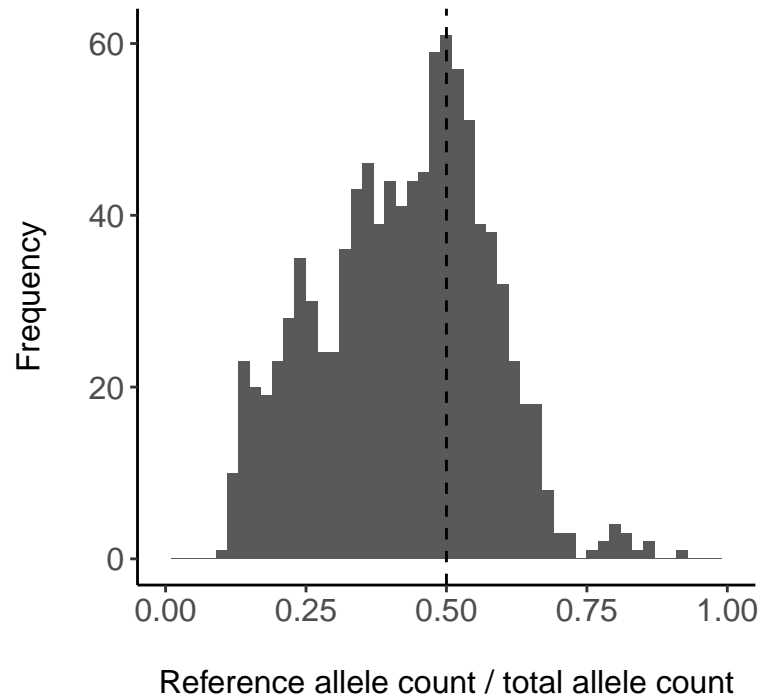*T. rathkei*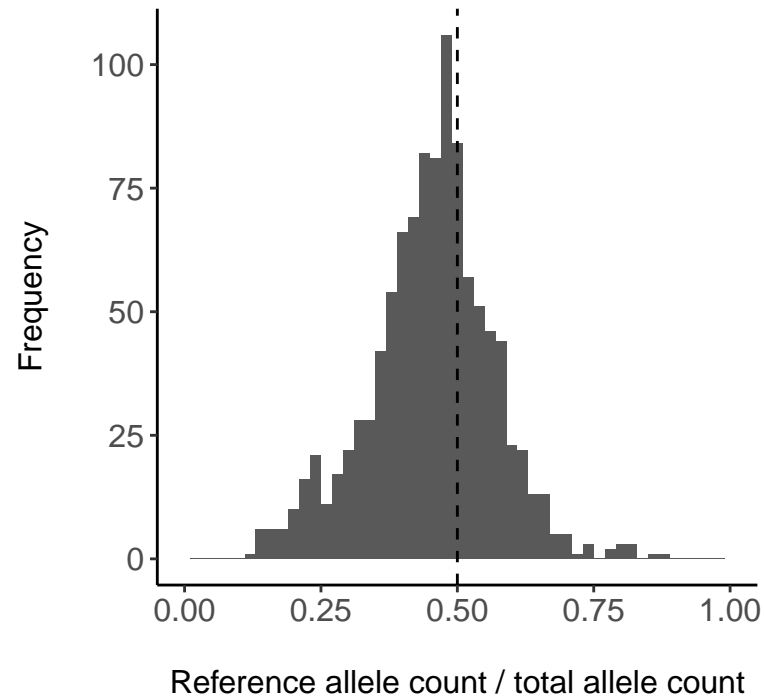

Supplement: Supplemental Information 2 [file peerj-12-17780-s002.zip › Yarbrough_Chandler_Code/5_snp_effects/5B_snp_similarity_and_allele_ratios/Figure1_onlybusco.pdf]
